# Editorial: Mapping the pathophysiology of schizophrenia: interactions between multiple cellular pathways, volume II

**DOI:** 10.3389/fncel.2023.1232677

**Published:** 2023-06-16

**Authors:** Natalie Matosin, Jess Nithianantharajah, Brian Dean, Chao Deng

**Affiliations:** ^1^Molecular Horizons, School of Chemistry and Molecular Bioscience, University of Wollongong, Wollongong, NSW, Australia; ^2^Florey Institute of Neuroscience and Mental Health, University of Melbourne, Parkville, VIC, Australia; ^3^Molecular Horizons, School of Medical, Indigenous and Health Sciences, University of Wollongong, Wollongong, NSW, Australia

**Keywords:** pathophysiology, schizophrenia, cellular, molecular, mechanism, neurotransmitters, antipsychotics

A decade ago, the first edition of “Mapping the Pathophysiology of Schizophrenia: Interactions Between Multiple Cellular Pathways” was curated by Deng and Dean (2013a). This Research Topic was highly successful, with contributing articles accessed over 260,000 times, and the Topic also published as a book (Deng and Dean, 2013b). The breadth of the topics in Volume I was (and remains) reflective of the complexity of schizophrenia and the challenges in fully elucidating its pathophysiology. It discussed the role of major neurotransmitter systems and their metabolism, key proteins implicated in the molecular pathology of schizophrenia, and the neurodevelopmental and vitamin D hypotheses of schizophrenia.

Many of the hypotheses and mechanisms discussed then remain highly relevant today, but technological advances have progressed the way we examine schizophrenia etiology and pathophysiology. For example, the accessibility of high-throughput “omics” methods have enabled collaborative networks like the Psychiatric Genetics Consortium Schizophrenia Working Group to comprehensively probe the genetics of schizophrenia and other psychotic disorders. The estimated heritability of schizophrenia is 60–80% (Monaco et al., 2018). With data on over 75,000 participants, there has been new insights into the genetic architecture of schizophrenia highlighting its polygenic nature with common variants involving over 280 genomic loci (Trubetskoy et al., 2022).

However, much work is still needed to fully elucidate the molecular pathology of schizophrenia. This requires not only extending the genetic studies, but given genetics alone does not explain the full risk profile of schizophrenia, investigating environmental factors and their downstream consequences outside of traditionally studied neurotransmitter systems (Misiak et al., 2018). This is particularly important given the growing understanding that environmental factors affect gene transcription and translation through epigenetic mechanisms (Ozomaro et al., 2013) and the downstream consequences of these gene x environmental interactions can be more readily moderated, thus may provide new opportunities for intervention.

In Volume II of this Research Topic, we have brought together a collection of articles that highlight the growing understanding of the molecular pathology of schizophrenia during brain development and later in life. Specifically, this Topic covers alterations of specific proteins and pathways that hold promise for future drug development to address schizophrenia symptomatology.

Fujihara firstly discusses progress in understanding the GABAergic hypothesis of schizophrenia from rodent models that capture GABAergic deficits. The article also describes an emerging connection between GABAergic signaling with microglia and neuroinflammation, presenting a novel mechanism by which GABA signaling may be impacted with disease progression. In accordance, the study by Su et al. demonstrates that neuroinflammation during development (via the rodent Poly:IC model) has downstream effects on gene expression of GABA receptors along with glutamate and serotonin receptors. This association was modulated by histone acetylation, demonstrating a link between neurotransmission, neuroinflammation and epigenetic mechanisms. Extending this, the findings by Pan et al. illustrate the importance of the miR-25-3p micro-RNA in modulating the salt-inducible kinase 1 (SIK1) protein. This is significant as SIK1 is related to multiple functions such as macrophage activity, cytokine secretion, circadian rhythms, sleep and metabolism, that are all impacted in the pathophysiology of schizophrenia (Darling and Cohen, 2021). Both histone acetylation and micro-RNAs are specific epigenetic mechanisms of interest for therapeutic applications, and understanding their mechanisms can lead to more effective treatments.

In the review by Singer and Yee the role of the endogenous molecule adenosine in schizophrenia is described. Adenosine shows promise as a novel treatment target for schizophrenia as it modulates both the glutamate and dopamine systems and thus ties together two of the leading hypotheses of schizophrenia. Despite this potential, adenosine-targeting drugs have not yet been realized, as key information regarding the presence of adenosine deficiency or dysfunction in schizophrenia is missing. This fundamental knowledge is needed to inform the development of adenosine-based drugs that are highly specific and efficacious. As an example for how detailed understanding of molecular pathology can lead to breakthrough in drug treatments, Dean et al. details how deep understanding of muscarinic receptors in schizophrenia—with decades of converging evidence from postmortem human, rodent and human brain imaging studies—has led a coformulation of drugs that targets central and peripheral muscarinic receptors to give an acceptable therapeutic benefit to side-effect profile to allow its use in humans. Muscarinic receptor targeting drugs are likely to soon emerge as clinically available treatments for patients, following successful Phase II and III clinical trials (Paul et al., 2022). This muscarinic receptor story eloquently illustrates how the development of new effective treatment approaches relies heavily on comprehensive understandings of specific pathophysiology.

Another necessary avenue of schizophrenia research is understanding the processes by which currently available antipsychotics operate, of which the mechanisms of action are still largely unknown. To this end, Hribkova et al. use two-dimensional neuronal cultures derived from individuals with schizophrenia known to respond to clozapine to extensively characterize the clozapine response in glutamatergic neurons. Glutamatergic neurons from individuals with schizophrenia showed reduced intrinsic electrophysiological properties, synaptic function and network activity, putatively by dysregulation of genes related to sodium and potassium channels as well as glutamate receptors. Interestingly, these electrophysiological and gene expression deficits could be restored with clozapine treatment. Importantly, this study shows pathophysiological deficits in schizophrenia patients could be modeled in the dish, which is immensely important for personalized treatment development efforts. However, the window in which treatment or intervention is given is critical to understand, yet commonly understudied. In the preclinical study by Visini et al. the antipsychotic effects of cannabidiol during adolescence in rodents was examined. The study concluded that while indirectly modulating the major neurotransmitter systems such as glutamate and GABA can be an effective approach, that timing of the intervention is likely to be key.

A decade on, the schizophrenia research field continues to pursue new approaches and targets to improve how we understand and treat schizophrenia. Volume II highlights that comprehensive understandings into the pathophysiology of schizophrenia, particularly deep mechanistic pathologies derived from gene x environment interactions, is essential. This knowledge is critical for developing new, effective, personalized treatment options with reduced side effects for those living with schizophrenia.

## Author contributions

NM drafted the editorial. All authors finalized the manuscript and approved the submission.
